# Periarticular blast wounds without fracture a prospective case series

**DOI:** 10.1186/s13018-024-04598-y

**Published:** 2024-02-06

**Authors:** Dana C. Covey, Christopher E. Gentchos

**Affiliations:** 1 Study Performed at University of California, San Diego, CA USA; 2 Naval Medical Center, San Diego, CA USA; 3 Level 2 United States Marine Corps Surgical Company, Al Anbar Province, Iraq; 4 Department of Orthopaedic Surgery, University of California, 200 West Arbor Drive, San Diego, CA 92103 USA; 5 Concord Orthopaedics PA, 264 Pleasant Street, Concord, NH 03301 USA

**Keywords:** Blast, Fragment, Trauma, Periarticular, Articular, Extremity

## Abstract

**Background:**

During the wars in Afghanistan and Iraq most injuries to service members involved the musculoskeletal system. These wounds often occurred around joints, and in some cases result in traumatic arthrotomy—a diagnosis that is not always clear, especially when there is no concomitant articular fracture. The aim of the present study is to evaluate the diagnosis and treatment of peri-articular blast injuries without fracture.

**Methods:**

The study cohort included 12 consecutive patients (12 involved extremities) who sustained peri-articular blast wounds of the extremities without fractures. The diagnosis of penetrating articular injury was based on clinical examination, radiographic findings, or aspiration. A peri-articular wound was defined as any wound, or radio-opaque blast fragment, within 5 cm of a joint. The New Injury Severity Score (NISS) was calculated for each patient. Four patients had upper, and 8 patients had lower extremity injuries. Nine of 12 patients had joint capsular penetration and underwent joint irrigation and debridement.

**Results:**

Two patients had retained intra-articular metal fragments. One patient had soft tissue blast wounds within 5 cm of a joint but did not have joint capsule penetration. There were no significant differences (*p* = 0.23) between the distribution of wounds to upper versus lower extremities. However, there were a significantly greater number of blast injuries attributed to Improvised Explosive Devices (IEDs) than from other blast mechanisms (*p* = 0.01).

**Conclusion:**

Extremity blast injuries in the vicinity of joints involving only soft tissues present a unique challenge in surgical management. A high index of suspicion should be maintained for joint capsular penetration so that intra-articular injuries may be appropriately treated.

## Introduction

During the wars in Afghanistan and Iraq, most injuries to U.S service members involved the musculoskeletal system [1, 2]. These injuries often occurred around one or more joints and may or may not have involved traumatic arthrotomy, a differential diagnosis that is not always straight forward. Previous reports have stressed the importance of treating intra-articular wounds [3, 4], and that an air-fluid level in the joint or intra-articular metal fragment was indicative of joint penetration requiring thorough irrigation [5]. Also suggested was synovial layer closure at the time of initial surgery if there is adequate tissue and no marked contamination, and delayed closure of fragment wounds [6]. However, in some cases, joint capsule penetration is not obvious, especially when there is no concomitant articular fracture. The aim of the present study is to specifically evaluate the diagnosis and treatment of peri-articular blast injuries without fracture.

## Patients and methods

This study was approved by the Institutional Review Board of Naval Medical Center, San Diego. Data were prospectively collected by a U.S. Marine Corps forward surgical team in Al Anbar Province, Iraq, that received casualties from the point of injury over a six-month period. This team was part of a Level 2 treatment facility within a 5-level echelon system where a higher number denotes increased sophistication of patient care. [7]

**Fig. 1:**
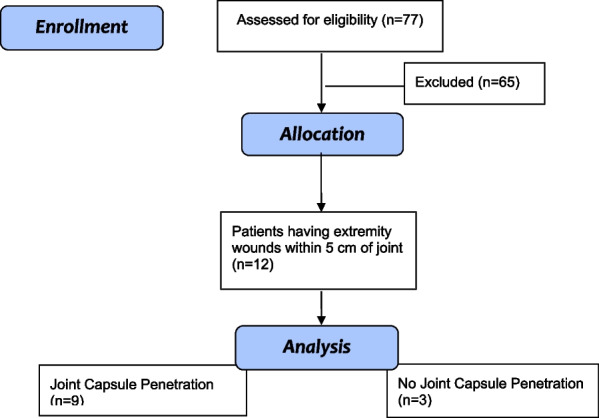
Patient flow diagram

From a cohort of 77 patients treated for predominantly battlefield blast and fragment injuries, 12 patients (12 involved extremities) were identified who met inclusion criteria for this study. These criteria were peri-articular blast wounds of the extremities without fracture, and any wound, or radio-opaque blast fragment within 5 cm of an upper or lower extremity joint (Fig. 1). Four study patients had upper extremity injuries and 8 patients had lower extremity injuries. All patients were male with mean age of 23 years (range, 20–30 years). Patient demographics, interventions, operative findings, and disposition were documented (Table 1). The Abbreviated Injury Scale (AIS) [8] was assigned to all injuries, and then New Injury Severity Score (NISS) [9] was calculated for each patient. Each casualty received intravenous antibiotics on presentation. Nine of 12 patients had joint capsular penetration and underwent joint irrigation. The diagnosis of articular injury was based on clinical examination, radiographic findings, and aspiration. Previously recommended criteria were used to help guide the choice to operate on soft tissue blast wounds. [10]

**Table 1:** Patient characteristics, injury, diagnostic and treatment data

Case	Age	Mechanism	Triage	Exam	Radiographs	Wounds	Diagnostic procedures	OR Procedures	Other injuries	AIS	NISS	Disposition
1	24	IED	Delayed transfer	Large effusion with painful R knee	Air-fluid level R knee, no fx	10 cm wound R knee	R knee hemarthrosis aspiration, positive saline load test	I&D, wound exploration, extended traumatic arthrotomy; irrigation, capsular closure	No other injuries R knee	2	4	Returned home
2	20	Mortar	Immediate	L ulnar nerve paresthesias	No intra-articular fragments, no effusion, no fx	2 Medial L elbow wounds (largest 2.5 cm) within 4 cm of medial epicondyle	None	I&D L elbow wounds, negative aspiration, and reverse arthrocentesis	No other injuries L elbow	2	4	Medevac Level 3
3	21	IED	Immediate	L ulnar nerve paresthesias	Metal fragment 8 cm distal to L elbow, no evidence of effusion, no fx	2 × 1 cm medial elbow wound 3 cm proximal to medial epicondyle	None	I&D of wounds, extension traumatic arthrotomy, capsular closure after irrigation	No other injuries L elbow	2	4	Medevac Level 3
4	21	Exploding rounds	Immediate	Large effusion, painful L knee	Multiple fragments L knee, none intra-articular, no fx	Multiple small wounds < 1–2 mm	Preoperative aspiration hemarthrosis	Arthrotomy, joint irrigation, primary closure	No other injuries L knee	2	4	RTD 3 weeks post injury
5	27	IED	Delayed	L knee pain	No retained fragments, no fracture	4 cm lateral wound at level of mid-patella L knee	Hemarthrosis aspirated L knee, positive saline load test	I&D, wound exploration, L knee arthrotomy, capsular closure after joint irrigation; delayed wound closure	R forearm soft tissue wound 2.5 cm w/o fracture	R forearm (1), L knee (2)	5	Medevac US
6	30	IED	Immediate	R hand pain	Metal fragment in 2nd MCP joint R hand, no fracture	1 cm wound dorsal index MCP joint	None	Exploratory laparotomy, I&D, wound exploration, joint capsule penetrated, arthrotomy extended, capsule closed after irrigation	Liver injury, soft tissue wounds R arm	Liver (3), R arm (1), hand (1)	11	Medevac Level 3
7	22	IED	Immediate	Diffuse pain consistent with injury	Metal fragment 5th MTP joint L foot, no fracture	1 cm lateral wound over 5th MTP joint	None	I&D wounds, traumatic arthrotomy extended, capsule closed after joint irrigated, delayed closure	Liver injury; soft tissue wounds R arm	Liver (2), R leg (1), R foot (1)	6	RTD 2 weeks post injury
8	25	VBIED	Immediate	Pain consistent with L knee injury	No retained fragments, effusion or fx on XR	3.5 cm lateral wound at proximal patella L knee	None	I&D wounds, joint capsule penetration, extension of arthrotomy, capsular closure after joint irrigation	No other injuries L knee	2	4	Medevac Level 3
9	20	Grenade	Minimal	L elbow joint irritability	Retained fragment intra-articular L elbow, no fx	2 mm posterior lateral wound	None	I&D L elbow wounds, removal intra-articular metal fragment, closure 1 cm joint capsule penetration after irrigation	No other injuries L elbow	2	4	Medevac Level 3
10	20	IED	Minimal	R knee peroneal nerve palsy, no effusion	Metal fragment, lateral to joint, no fx	1 × 2 cm wound, just proximal to fibular head, dense motor and sensory peroneal nerve palsy	None	Negative R knee aspiration, large, retained metal fragment, wound exploration, longitudinal rent in otherwise intact peroneal nerve, wounds irrigated, extension closed	No other injuries R knee	2	4	Medevac Level 3
11	24	Grenade	Minimal	Pain L ankle joint	Few retained metal fragments anterior ankle, suspicious for joint penetration	1 × 1 cm ant-lat and 1 × 2 cm ant-med	None	I&D, wound exploration, anterior capsule rent 2 cm, joint irrigated and closed, wound extension closed	Superficial wounds both shoulders	AIS = (1), left leg (1) left ankle = (2) moderate	5	Medevac Level 3
12	22	IED	Delayed	No L knee effusion, minimal pain, near full ROM	Retained metal fragment level of superior pole patella, no fx	1 mm wound superior pole L patella	None	IV antibiotics	Small fragment wounds	R leg AIS = (1)minor; L leg AIS = (1) minor; R knee AIS = (1) minor	2	RTD 2 weeks post injury

IED—Improvised explosive device; VBIED—vehicle borne IED; R—right; L—left; Fx—fracture; ROM—range-of-motion; XR—radiograph; MCP—metacarpal phalangeal; Ant-lat—anterolateral; Ant-med—anteromedial; OR—operating room; AIS—abbreviated injury scale; NISS—new injury severity score; Medevac—medical evacuation; RTD—returned to duty

For standardization purposes, we defined an operative case as any patient anesthetized for surgery. A procedure was defined as any operation performed on a single extremity, or any operation performed in the abdomen, chest, head, or neck. Multiple blast wounds requiring surgical treatment in a single extremity were logged as a single procedure.

A surgical arthrotomy was typically performed by extending the existing traumatic wound. Wound debridement was performed by excising skin sparingly, excision of subcutaneous fat and fascia excision as needed, and excision of devitalized muscle tissue.

Foreign bodies were removed unless they were far from the missile path, and their removal did not cause significant additional soft tissue injury. Articular capsules and synovium were sparingly excised to facilitate closure after primary surgical treatment [11, 12]. All wounds were irrigated with an isotonic solution and not closed primarily.

### Statistical analysis

Descriptive statistics were used for demographic data. The Chi-square test was used for statistical testing with significance set at *p* < 0.05.

## Results

All patients in this study received general anesthesia, and none required blood transfusion as part of their resuscitation. There were no patient deaths. Two patients had retained intra-articular metal fragments. There was one patient that had soft tissue blast wounds within 5 cm of a joint that did not penetrate the joint capsule or meet criteria for surgery. There were no significant differences (*p* = 0.23) between the distribution of wounds to upper versus lower extremities. However, there were a significantly greater number of blast injuries attributed to Improvised Explosive Devices (IEDs) than from other blast mechanisms (*p* = 0.01; Table 2). There were no immediate postoperative wounds infections.

**Table 2:** Mechanisms of injury

Mechanism	No. of patients
Improvised explosive device	8
Grenade	2
Exploding rounds	1
Mortar	1

**Fig. 2:**
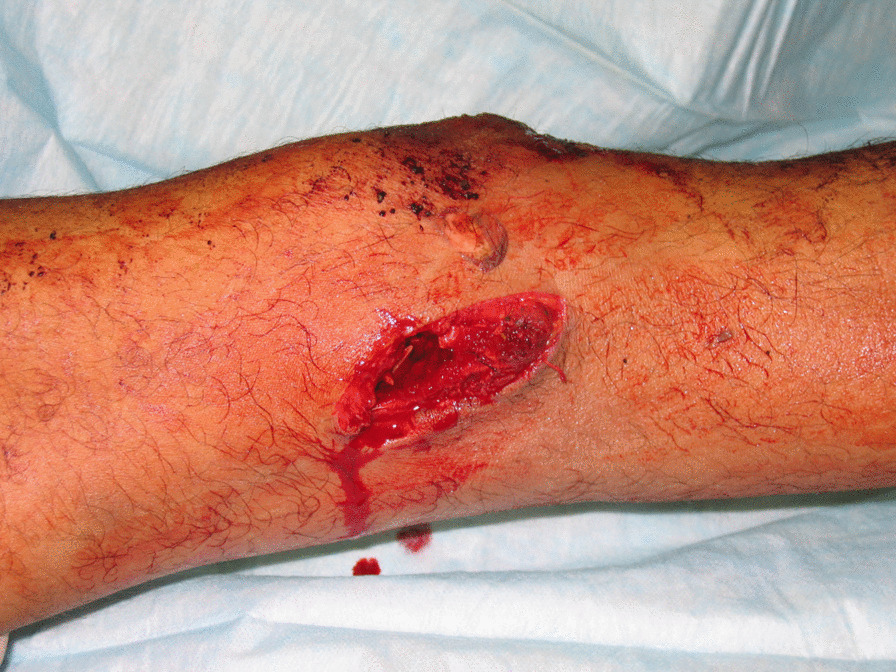
This 24-year-old male presented with a painful right knee and soft tissue IED blast wounds

Two cases highlight the diagnostic challenges when trying to determine whether a peri-articular wound has penetrated the joint capsule. Case 1, a 24-year-old patient, was transferred to our facility with a painful right knee after having undergone previous surgical treatment of soft tissue IED blast wounds on the date of injury (Fig. 2). Antibiotics had been administered since the index injury. He subsequently developed severe pain, and a hemorrhagic effusion confirmed by preoperative arthrocentesis. He was taken to the operating room where a right knee arthrotomy was performed. Surgical findings showed there was an extra-articular blast wound, but a missed posterior knee joint capsular injury that was corroborated by a saline load test. The joint was thoroughly irrigated and then closed over a drain. He was discharged without further complications.

**Fig. 3:**
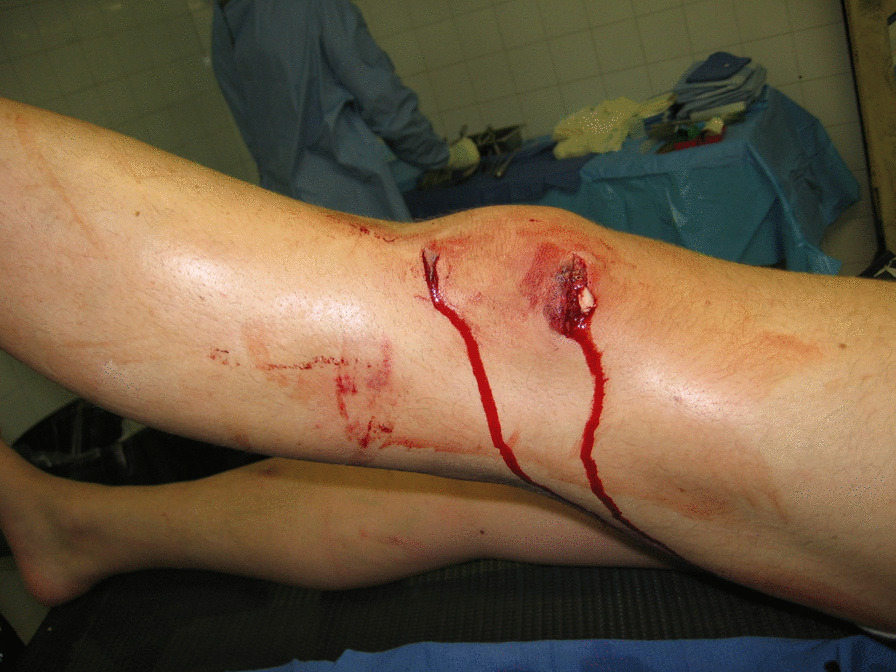
This 21-year-old male sustained soft tissue injury to the left knee region from an explosive blast

Case 4, a 21-year-old service member, presented with a blast injury to the left knee (Fig. 3). Radiographs showed retained metal fragments, none of which appeared intra-articular. There was no air-fluid level on the initial radiographs, and initially none of the wounds met standard criteria for surgical management. The patient was initially treated according to recommendations for non-operative treatment of blast wounds that included 24 h of intravenous antibiotics (cephazolin and clindamycin) followed by 4 days of oral antibiotics [13–15]. However, 4 days after injury he developed severe pain, and hemorrhagic effusion confirmed by preoperative joint aspiration. Arthrotomy and thorough joint irrigation and debridement resulted in full resolution of symptoms and return to full duty 3 weeks after injury.

## Discussion

This series suggests that blast wounds located near joints require special consideration for optimal treatment, including a low threshold for diagnostic arthrocentesis. Depending upon whether there was damage to the articular capsule, joint injuries were classified as penetrating or nonpenetrating [4]. Suspected penetrating injuries of joints should be treated immediately, and arthrotomy performed if there is a high index of suspicion or confirmation that penetration occurred [16]. The joint should be copiously irrigated, debrided of foreign material and nonviable tissue, and drained, with primary closure of the synovial layer (if possible) and delayed primary closure of the skin; or alternatively, use of skin grafts or flaps [3, 17]. Nikolic et al. [3] presented their results of treatment of war injuries involving major joints in 339 patients, 176 (51.9%) of whom were injured by high explosive fragments. Early complications occurred in seventy-seven (22.7%) of the patients. Thirty-two (9.4%) had either joint or soft tissue infection, and eighty-one (23.9%) required subsequent reconstructive surgery.

There is a paucity of literature specifically addressing the evaluation and treatment of peri-articular blast injuries without associated fractures. These wounds are more challenging to diagnose compared with peri-articular blast injuries that cause intra-articular fracture because the latter facilitate radiographic diagnosis of capsular penetration. However, radiographic findings of an air-fluid level in the joint, or the presence of intra-articular metal fragments indicate joint penetration requiring arthrotomy, debridement and thorough irrigation [4, 13]. An attempt at synovial layer closure at the time of initial surgery is reasonable with delayed closure of more superficial layers [4]. While intra-articular penetration is obvious in many cases, four cases in this series were not readily apparent. While an acute traumatic hemorrhagic effusion itself can cause severe pain and joint irritability, so can infection in those patients sustaining penetration of a joint by blast fragments.

The cases presented here highlight several important points. First, joint capsule penetration by blast fragments introduces bacteria into the joint and usually cannot be managed with antibiotics alone. Second, guidelines for non-operative management suggested by Bowyer [13] included wounds affecting soft tissue only (no fractures, no breach of pleura or peritoneum and no major vascular involvement), a wound entry or exit less than 1–2 cm in maximum dimension, no evidence of cavitation, and not infected. These non-operative treatment criteria should also exclude suspected or actual joint penetration. The absence of an air-fluid level or metal fragments in a joint also does not exclude capsular violation. Finally, if the diagnosis is not clear at the time of presentation, then close follow-up is needed to ensure prompt treatment as soon as the full extent of injury declares itself. This final point is important in a military system of rapid transfer to the next echelon care.

Questionable cases of capsular penetration should be corroborated by aspiration of blood from the joint, or by a positive saline load test that involves intra-articular injection of sterile saline (60 mL for the knee). The wound in question is observed for evidence of leakage at rest, and with joint passive range-of-motion—a procedure that is often not conclusive [18, 19]. Tornetta et al. [19] found this test to have a sensitivity of only 43% for small arthrotomies of the knee and should not be used as the sole means to identify an open knee injury. When the result of this test is negative, then other factors, such as patient symptoms, painful joint movement, the extent of soft tissue damage, and a fragment’s trajectory as suggested on radiographs should be carefully considered. Advanced imaging such as CT, although not available in our field setting, would be a useful adjunct to diagnosis. The decision to perform a surgical arthrotomy rests with the surgeon’s best judgement considering the mechanism of wounding, history and physical examination, radiographic findings and aspiration or fluid injection. This series suggests that in treating blast injured patients with soft tissue wounds near joints, a careful search for intra-articular penetration should occur even if there are no articular fractures appreciated clinically or radiographically.

## Conclusion

Peri-articular blast wounds involving only soft tissues can present a diagnostic challenge for optimal management. A high index of suspicion should be maintained for joint capsular penetration in treatment decisions for peri-articular soft tissue wounds without fractures. This series suggests that aspiration of blood from the joint may be an indication for operative management in these cases.

## Data Availability

No datasets were generated or analyzed during the current study.

## References

[1] Owens BD, Kragh JF, Wenke JC, Macaitis J, Wade CE, Holcomb JB (2008). Combat wounds in operation iraqi freedom and operation enduring freedom. J Trauma.

[2] Belmont PJ, McCriskin BJ, Hsiao MS, Burks R, Nelson KJ, Schoenfeld AJ (2013). The nature and incidence of musculoskeletal combat wounds in Iraq and Afghanistan (2005–2009). J Orthop Trauma.

[3] Nikolić D, Jovanović Z, Popović Z, Vulović R, Mladenović M (1999). Primary surgical treatment of war injuries of major joints of the limbs. Injury.

[4] Nikolic D, Drasœkovic V, Vulovic R, Mladenovic M (2000). Missile injuries of the knee join. Injury.

[5] Patzakis MJ, Dorr LD, Ivler D, Moore TM, Harvey JP (1975). The early management of open joint injuries. A prospective study of one hundred and forty patients. J Bone Joint Surg..

[6] Gafoor PM, Purushothaman R (2018). Open knee joint injury score (OKJIS)—a scoring system for open injuries of the knee joint-A pilot study. Keral J Orthop.

[7] Bagg MR, Covey DC, Powell ET (2006). Levels of medical care in the Global War on Terrorism. J Am Acad Orthop Surg.

[8] Greenspan L, McLellan B, Greig H (1985). Abbreviated injury scale and injury severity score: a scoring chart. J Trauma.

[9] Osier T, Baker S, Long W (1997). A modification of the injury severity score that both improves accuracy and simplifies Scoring. J Trauma.

[10] Covey DC (2002). Blast and fragment injuries of the musculoskeletal system. J Bone Joint Surg..

[11] Barr RJ, Mollan RA (1989). The orthopaedic consequences of civil disturbance in Northern Ireland. J Bone Joint Surg Br.

[12] Stanec Z, Skrbic S, Dzepina I, Hulina D, Ivrlac R, Unusic J, Montani D, Prpic I (1993). High-energy war wounds: flap reconstruction. Ann Plast Surg.

[13] Bowyer GW (1997). Management of small fragment wounds in modern warfare: A return to Hunterian principles?. Ann R Coll Surg Engl.

[14] Covey DC, Lurate RB, Hatton CT (2000). Field hospital treatment of blast wounds of the musculoskeletal system during the Yugoslav civil war. J Orthop Trauma.

[15] Hill PF, Edwards DP, Bowyer GW (2001). Small fragment wounds: biophysics, pathophysiology and principles of management. J R Army Med Corps.

[16] Covey DC, Peterson DA (1995). Treatment of musculoskeletal blast wounds at a Navy field hospital during the Balkans War. Tech Orthop.

[17] Madenwald MB, Fisher RC (1995). Experiences with war wounds in Afghanistan and Mozambique. Tech Orthop.

[18] Konda SR, Howard D, Davidovitch RI, Egol KA (2013). The saline load test of the knee redefined: a test to detect traumatic arthrotomies and rule out periarticular wounds not requiring surgical intervention. J Orthop Trauma.

[19] Tornetta P, Boes MT, Schepsis AA, Foster TE, Bhandari M, Garcia E (2008). How effective is a saline arthrogram for wounds around the knee?. Clin Orthop Relat Res.

